# Fluorescent Powassan Reporter Viruses Infect Neuron, Astrocyte and Microglial Cell Lines Independent of Attenuating D308N Envelope Protein Modification

**DOI:** 10.3390/v18070768

**Published:** 2026-07-13

**Authors:** Autumn Y. Laird, Varvara Kirillov, Elena E. Gorbunova, Alexander Vostrov, Genevieve Rochlin, Catherine E. Finnerty, Aisling G. Byrne, Priscila Ikeda, Romario Matos, Marissa R. Lindner, Hwan Keun Kim, Erich R. Mackow

**Affiliations:** 1 Department of Microbiology and Immunology, Center for Infectious Disease, Renaissance School of Medicine, Stony Brook University, Stony Brook, NY 11794-5222, USA; autumn.laird@stonybrook.edu (A.Y.L.); varvara.kirillov@stonybrook.edu (V.K.);; 2 Department of Microbiology and Immunology, Renaissance School of Medicine, Life Sciences Rm 126, Stony Brook University, Stony Brook, NY 11794-5222, USA

**Keywords:** Powassan virus, fluorescent reporter virus, attenuated mutant, NS1 deletion, Envelope EDIII mutant, BSL2 POWV, glial cells, neurons

## Abstract

The Powassan virus (POWV) is a neurovirulent tick-borne virus that causes age-associated lethality and long-term neurologic sequelae in 50% of survivors. In aged mice the POWV strain LI9 mirrors human lethality and neuropathology; however, an avirulent POWV mutant, LI9-D308N, fails to enter the CNS or cause lethal disease. The D308N mutation is present in an envelope protein domain associated with cell attachment, yet the role of D308N mutations in cell tropism and neuroinvasion remains to be resolved. Here, we engineered fluorescent mScarlet3 and mNeonGreen reporter genes into WT LI9, and avirulent LI9-D308N viruses and assessed their ability to infect CNS cells in vitro. In addition, we generated replication-defective reporter POWVs that only replicate in NS1-expressing cells by replacing NS1 with fluorescent genes. Similar to WT LI9, fluorescent reporter POWVs spread focally and nonlytically, are stable following passage and reach high titers 2–5 dpi. In NS1-expressing VeroE6 cells, LI9-ΔNS1-FL reporters exhibited robust fluorescence 24 h post-infection (hpi), while fluorescence from LI9-reporter infections was first observed ~32 hpi. Comparing LI9-mScarlet3 and avirulent LI9-D308N-mScarlet3 viruses revealed no difference in their ability to infect human brain microvascular endothelial cells, pericytes, astrocytes, microglia or neuronal cells in vitro. Notably, LI9-mScarlet3 viruses productively and persistently infected differentiated, neuron-like, SH-SY5Y cells without apparent cytotoxicity. These findings indicate that LI9-D308N is capable of infecting blood–brain-barrier and CNS cells, and suggest that neuroinvasion is restricted prior to LI9-D308N engaging CNS cells. These results are consistent with clearance of LI9-D308N from the blood, or the D308N mutation interfering with potential routes of POWV neuroinvasion. Collectively, fluorescent POWV reporter viruses provide insight into the mechanism of POWV neuroinvasion, permit analysis of replication-defective POWVs as vaccines and provide a means of analyzing POWV cell tropism, antivirals and cell-to-cell spread in BSL2 and BSL3 settings.:

## 1. Introduction

Orthoflavivirus (OFV) is a genus in the *Flaviviridae* family that is predominantly comprised of arthropod-borne positive-strand RNA viruses that cause diverse human diseases [1]. Mosquito-transmitted orthoflaviviruses (OFVs) include yellow fever, dengue, Zika, West Nile and Japanese encephalitis viruses (YFVs, DVs, ZVs, WNVs, JEVs), while various tick-transmitted OFVs include neurotropic tick-borne encephalitis virus (TBEV) and Powassan virus (POWV) [1,2,3,4,5,6]. The geographic range of vectors, hosts and their discrete zoonotic cycles delimit OFV transmission to humans [7,8,9,10,11,12,13]. OFVs cause infections ranging from asymptomatic to severe acute disease, with clinical outcomes shaped by both host and viral determinants, including age, immune status, prior exposure history, viral genotype and tissue tropism [1,2,3,7,14,15]. POWVs were first isolated in 1958 from a 5-year-old boy with fatal encephalitis [16]; however, >80% of clinical POWV infections are observed in individuals over 40, with fatalities restricted to individuals >49 years of age [15,17,18]. POWV disseminates from tick bite sites to the CNS, and in severe cases causes acute encephalitis that is 10% fatal and results in long-term neurologic deficits in ~50% of survivors [2,17,19]. However, a limited number of human POWV cases have been investigated, primarily at autopsy [14,15]. POWV spread to the CNS, neurotropism, age-dependent severity, pathogenic mechanisms and long-term neurologic deficits remain to be determined and there are no approved vaccines or therapeutics to prevent or resolve the severity of POWV infections [2].

POWV is the only known North American tick-borne OFV [1,2]. There are two POWV genetic lineages (lineage I and lineage II—deer tick virus) that differ in their primary mammalian hosts and tick species, but which share 96% identical envelope (Env) proteins and comprise a single POWV serotype [9,20,21,22]. OFV genomes (~11 kb) encode a polyprotein comprised of three structural and seven non-structural (NS) proteins that are co-translationally cleaved by signal peptidases, and cellular and viral proteases [1]. An N-terminal cytoplasmic capsid (C) protein is followed by ER-localized prM and Env structural proteins. Downstream NS proteins are threaded across ER membranes leaving proteins required for RNA synthesis within the ER, across ER membranes and in the cytoplasm [1]. The viral protease, NS2B3, is formed by integral ER membrane protein NS2B in complex with the cytoplasmic NS3 protease/helicase protein [1]. NS1 proteins are ER-translocated yet NS1 organizes cytoplasmic RNA transcription complexes and is required for OFV replication [1,23]. RNA-capsid protein assembly nucleates virion budding into the ER lumen and acquisition of viral surface proteins prM and Env [1,24,25]. Within the trans-Golgi furin cleavage of immature virion Env trimers, Env is reorganized into 90 antiparallel dimers that form an isocahedral shell that surrounds OFV lipid bilayers [26,27,28].

LI9 is a lineage II deer tick-derived POWV isolate that causes age-dependent lethality and neurovirulent disease in immunocompetent C57Bl/6 mice [29,30], mirroring age-dependent human disease severity. In mice, peripheral inoculation with a minimal infectious dose of LI9 causes lethal encephalitis with prominent neurologic sequelae [29,31]. Persistent passage of LI9 in VeroE6 cells resulted in an avirulent POWV, LI9P, which failed to cause neurologic signs, symptoms or lethality in B6 mice [31]. Sequencing revealed a single D308N mutation in domain III of the LI9P envelope protein (EDIII) [31], a domain broadly associated with OFV virulence, cell attachment and attenuation [4,28,31,32,33,34,35,36,37,38,39,40,41,42,43,44,45]. Reverse genetic D308N mutation alone abolished LI9 neurovirulence, lethality and neuroinvasion of the CNS [31]. Despite this, LI9P and LI9-D308N viruses elicit immune responses that protect mice from a WT POWV challenge [31]. However, mechanisms by which EDIII D308N residues restrict POWV spread to the CNS remain to be resolved.

Reverse genetics has permitted the modifying of recombinant POWVs, and thereby, the analysis of virulence determinants and protein functions that inform pathogenic mechanisms and vaccine development [46,47]. The development of recombinant OFVs that express luciferase or fluorescent reporter proteins have facilitated studies of OFV dissemination, cell entry, tropism and attenuation [48,49,50,51,52,53,54,55,56,57,58]. However, the stability of foreign reporter genes inserted into recombinant OFVs varies, often requiring position-specific insertion, partial sequence scrambling and T2A/P2A translational skipping cassettes in order to direct polyprotein synthesis and still maintain OFV replication [50,51,52,58]. Reporter OFVs with NS1 protein deletions are rescued by NS1 expression *in trans*, and are replication-defective in the absence of NS1 [23,41,46,47,51,54,59]. As a result, OFV-ΔNS1 viruses are nonpathogenic BSL2-compatible viruses with vaccine potential [46,47].

Here we used POWV LI9 reverse genetics [54] to develop both BSL2- and BSL3-compatible fluorescent reporter POWVs and analyze POWV infection of CNS cells in vitro. We created POWV NS1-expressing VeroE6 cell lines required for the replication of NS1-deleted LI9 viruses. Circular polymerase extension reaction (CPER) reverse genetics [54] was used to generate infectious LI9-mScarlet3 [60], LI9-mNeonGreen [61] and LI9-D308N-mScarlet3 reporter viruses, as well as replication-defective LI9-ΔNS1-mScarlet3 and LI9-ΔNS1-mNeonGreen reporters. Analysis revealed that BSL3 restricted POWV LI9 and LI9-D308N mutant reporter viruses replicated to high titer in VeroE6 cells and that NS1 protein expression is required for LI9-ΔNS1-reporter virus replication. Both LI9-mScarlet3 and LI9-D308N-mScarlet3 reporter viruses similarly infected CNS associated endothelial, microglial, astrocyte, pericytes and differentiated SH-SY5Y neuronal cells [62,63,64] in vitro. These findings indicate that LI9-D308N mutants, which fail to enter the CNS in vivo [31], are fully capable of infecting blood–brain-barrier and CNS cell types in vitro. This suggests that LI9-D308N neuroinvasion is constrained peripherally, prior to engaging CNS barriers or cells, or blocked from entering the CNS by a discrete entry route. These findings rationalize analyzing POWV routes of CNS entry and the role of EDIII D308N mutations in neuroinvasion. Overall, our findings demonstrate the utility of fluorescent BSL2 and BSL3 POWVs in analyzing viral replication and cell tropism in vitro, and provide POWV reporters for use in antiviral screening and vaccine development.

## 2. Methods

### 2.1. Cells and Virus

VeroE6 (ATCC CRL 1586) were grown in Dulbecco’s modified Eagle’s medium (DMEM) with 8% FBS, and 1% antibiotic/antimycotic (Gibco, Waltham, MA, USA) at 37 °C in 5% CO_2_, as previously described [29,30,31,54]. POWV strain LI9 (GenBank accession number: MZ576219) was isolated from field-collected *Ixodes scapularis* collected from Long Island, NY, USA [30]. LI9 was inoculated onto VeroE6 cells and passaged 3–5 times in VeroE6 cells to generate stocks. POWVs were absorbed to 70% confluent VeroE6 cell monolayers. After 2 h, monolayers were PBS-washed and grown in DMEM-2% FBS. POWV titers were determined by duplicate focus assay and focus-forming unit (FFU) titers were analyzed using Prism 10 (GraphPad Software V5) [29,30,31,54]. Briefly, POWVs were serially diluted on VeroE6 and 30 hpi-infected foci were quantified by immunoperoxidase staining as indicated with anti-POWV capsid (1:2000) or anti-POWV hyperimmune mouse ascites fluid (HMAF; 1:4000; ATCC) [29,30,31,54]. All work with infectious POWVs was conducted in a certified BSL3 containment facility. Sequencing was performed by the SBU Genomics facility, as previously described [29,30,31,54].

Human neuroblastoma cell line SH-SY5Y (ATCC CRL 2266) was grown in DMEM/F-12 (Gibco) supplemented with 10% fetal bovine serum (FBS) and 1% antibiotic antimycotic solution (Sigma-Aldrich; St. Louis, MO, USA) [62,63,64]. To yield mature neuronal-like cells, SH-SY5Y cells were differentiated for two weeks with retinoic acid (RA, 10 µM) and brain-derived neurotrophic factor (BDNF) (StemCell Technologies; Vancouver, BC, Canada) [62,63,64,65]. SH-SY5Y cells were treated with RA on days 0, 3 and 5 in 10% DMEM/F-12 supplemented with 10% FBS and 1% antibiotic antimycotic solution. On days 7, 10 and 14 the SH-SY5Y cells were treated with 10 μg/mL BDNF in Neurobasal Plus media (Gibco) supplemented with 2% B-27 (Gibco), 1% antibiotic antimycotic solution and 1% GlutaMAX (Gibco) [62,63,64].

Murine astrocyte cell line, C8-D1A (CRL-2541), was grown in DMEM (Dulbecco’s modified Eagle’s medium) supplemented with 10% fetal bovine serum, and antibiotic antimycotic solution. Murine microglial cell line, SIM-A9 (CRL-3265), was grown in DMEM/F-12 supplemented with 10% fetal bovine serum and 1% antibiotic antimycotic solution. Primary human pericytes, HBVP (ScienCell #1200; Carlsbad, CA, USA), were grown in Pericyte Growth Medium 2 (PromoCell C-28041; Heidleberg, Germany) supplemented with SupplementMix (PromoCell C-39841) and antibiotic antimycotic solution. Primary human brain microvascular endothelial cells (hBMECs) were purchased from Cell Biologics (H-6023), grown in EC basal medium-2 MV (EBM-2 MV; Lonza; Basel, Switzerland) 10% FBS, and 1% antibiotic antimycotic solution supplemented with EGM-2 MV SingleQuots (Lonza), and incubated at 37 °C and 5% CO_2_. All cells were maintained at 37° with 5% CO_2._

All infections with LI9-mScarlet3 and LI9-mNeon were conducted in a certified BSL3 facility at Stony Brook University. SH-SY5Y cells were inoculated with LI9-mScarlet3 in DMEM/F-12 with 2% fetal bovine serum with a two-hour absorption period at 37 °C and 5% CO_2_. After absorption, the SH-SY5Y monolayer was washed with phosphate-buffered saline (PBS) and maintained in DMEM/F-12 with 10% FBS. SIM-A9 cells were similarly infected and maintained in DMEM/F-12 containing 2% FBS after infection. C8-D1A cells and VeroE6 cells were infected in DMEM containing 2% FBS. HBVPs were infected in DMEM with 2% FBS and subsequently replenished with Pericyte Medium with SupplementMix after absorption. HBMECs were infected in DMEM supplemented with 2% FBS and maintained after in endothelial-cell basal medium-2 MV supplemented with EGM-2 MV SingleQuots (Lonza; Basel, Switzerland).

### 2.2. Lentivirus Production and VeroE6 Transduction

NS1-Flag, NS1-HA and NS1-KDEL VeroE6 cell lines were generated using lentivirus transduction approaches, as previously described [29,30,54]. Briefly, lentivirus was generated using psPAX2 packaging plasmid #12260 (Addgene; Watertown, MA, USA), pMD2.G envelope plasmid #12259 (Addgene; Watertown, MA, USA) and transgene constructs generated in pLenti-puro plasmid #39481 (Addgene; Watertown, MA, USA). HEK293T cells were transfected with psPAX2, pMD2.G and pLenti-puro plasmids using polyethylenimine (PEI) in Opti-MEM. One day post-transfection, media were changed and replaced with DMEM containing 8% FBS and 1% antibiotic/antimycotic solution. Two days post-transfection, lentivirus-containing supernatants were collected and passed through a 0.45 uM low-protein-binding filter to remove cell debris. VeroE6 cells were subsequently transduced with lentivirus stocks and puromycin-selected (6 µg/mL). Expression of NS1-Flag, NS1-HA and NS1-KDEL was assessed using Western blot.

### 2.3. Western Blotting

Expression of NS1-Flag, NS1-HA and NS1-KDEL in VeroE6 cells was assessed through Western blotting. Transduced, infected or control VeroE6 cells were washed with PBS prior to cell lysis with buffer containing 50 mM Tris pH 7.4, 150 mM NaCl, 2 mM EDTA, 1% Triton X-100, protein inhibitor cocktail P8340 (MilliporeSigma; Burlington MA, USA) and 1 mM PMSF. Following cell lysis, protein levels were determined in a bicinchoninic acid assay (Thermo Scientific; Waltham, MA, USA) and 10 µg of protein was resolved with SDS–12% polyacrylamide gel electrophoresis. Proteins were transferred to PVDF, blocked in 5% TBST non-fat dry milk, and incubated with the indicated antibodies at 1:1000. Antibodies used were anti-NS1 rabbit antibody (GeneScript; Piscataway NJ, USA) and Anti-β-Actin antibody A5441400 (MilliporeSigma). Proteins were detected using HRP-conjugated anti-mouse or HRP-conjugated anti-rabbit antibodies with Immobilon Crescendo Western HRP Substrate (Millipore, WBLUR0500). Western blot images were captured on an ImageQuant LAS 500.

### 2.4. Generation of POWV LI9 Fluorescent Reporter Constructs

pMiniT plasmids containing clones of POWV genome fragment 1–5 and linker fragments were previously described [31,54]. POWV fragments were PCR amplified using high-fidelity Phusion polymerase (NEB; Ipswich, MA, USA) and gel-purified and CPER-assembled prior to transfection of VeroE6 cells. Fluorescent reporter constructs were generated through NEBuilder HiFi DNA assembly (NEB; Ipswich, MA, USA). LI9 Fluorescent reporter genes mScarlet3 [60] or mNeonGreen [61] were inserted into F1 fragment pMiniT clones, 38 bp downstream of and in-frame with the POWV capsid protein, followed by a T2A translational skipping motif (EGRGSLLTCGDVEENPG-P) [66,67] and a scrambled capsid sequence using NEBuilder HiFi DNA assembly. Plasmids were sequenced and reporter-inserted F1-FL fragments were amplified, substituted into CPER reactions for WT F1 and Lipofectamine 3000 (Thermo Scientific) transfected into VeroE6 cells [31,54].

To generate NS1-deleted POWVs, pMiniT-F2 plasmids were subjected to site-directed deletional mutagenesis removing residues 5–297 or 158–255 in-frame with NS1 coding sequences. F2-ΔNS1 fragments were amplified and substituted for WT F2 fragments in CPER reactions to generate LI9-ΔNS1 viruses by transfection of NS1-expressing VeroE6 cells. Fluorescent LI9-ΔNS1 reporters were generated by HiFi DNA assembly of mScarlet3 or mNeonGreen genes by in-frame insertion into NS1-Δ158–255 residue deletion mutants. CPER reactions containing F2-ΔNS1-mScarlet3 or -mNeonGreen fragments in place of WT F2 were Lipofectamine 3000-transfected into NS1-expressing VeroE6 cells [31,54].

### 2.5. CPER Production of POWV Reporter Viruses

Infectious reporter POWVs were generated using CPER, as previously described [31,54]. Briefly, LI9 fragments F1-F5 and a UTR linker fragment, containing 26 nt overlapping regions, were amplified from pMiniT plasmids using an initial denaturation: 98 °C for 30 s, 32 cycles of 98 °C for 20 s, 60 °C for 30 s, and 72 °C with 30 s per kb, and a final extension at 72 °C for 5 min. Resulting fragments were gel-purified and Monarch kit (NEB; Ipswich, MA, USA)-extracted, and 0.1 pmol of each DNA fragment was CPER-circularized in a 50 µL reaction containing 200 µM of dNTPs, 1× Phusion polymerase GC reaction buffer, and 1 µL Phusion polymerase (NEB; Ipswich, MA, USA): initial denaturation 98 °C for 30 s, 12 cycles of 98 °C for 20 s, 60 °C for 30 s, and 72 °C for 10 min, and a final extension at 72 °C for 10 min. To generate infectious fluorescent POWV, circularized CPER constructs were transfected into VeroE6 cells using Lipofectamine 3000.

### 2.6. Immunofluorescence

Images of SH-SY5Y, C8-D1A, SIM-A9, HBVP, VeroE6 and hBMEC cells infected with LI9-mScarlet-3, LI9-D308N-mScarlet or LI9-mNeon were resolved on a Nikon eclipse TE2000-S microscope equipped with a PCO panda sCMOS camera (Nikon Corporation, Tokyo, Japan) and NIS-Elements software (V5.21.00) in BSL3 or an Olympus IX51 microscope with an DP71 camera. Images were taken multiple days post infection and representative images are shown. Overlaid images were generated using Fiji software (V2.16.0). Images of NS1-Flag-, NS1-HA- and NS1-KDEL-expressing VeroE6 cells infected with LI9-ΔNS1-mScarlet3 and LI9-ΔNS1-mNeonGreen were resolved on an Olympus IX51 microscope with an Olympus DP71 camera. Images were taken on a YFP channel and subsequently processed on Fiji software. Images of susceptible infected cells and bright field images were ImageJ-merged and quantified by manual counting of at least 5000 cells total from LI9-mScarlet infections and 3500 cells total from LI9-D308N-mScarlet infections.

### 2.7. Statistical Analysis

Statistics were performed using Prism 10 software (Graphpad Software Inc.). Viral titers in cell supernatants were assayed in duplicate and presented as the mean +/− SD analyzed by two-way ANOVA. Statistical analysis of each experiment is described in corresponding figure legends.

## 3. Results

### 3.1. Generation of Fluorescent LI9 POWVs

**Figure 1:**
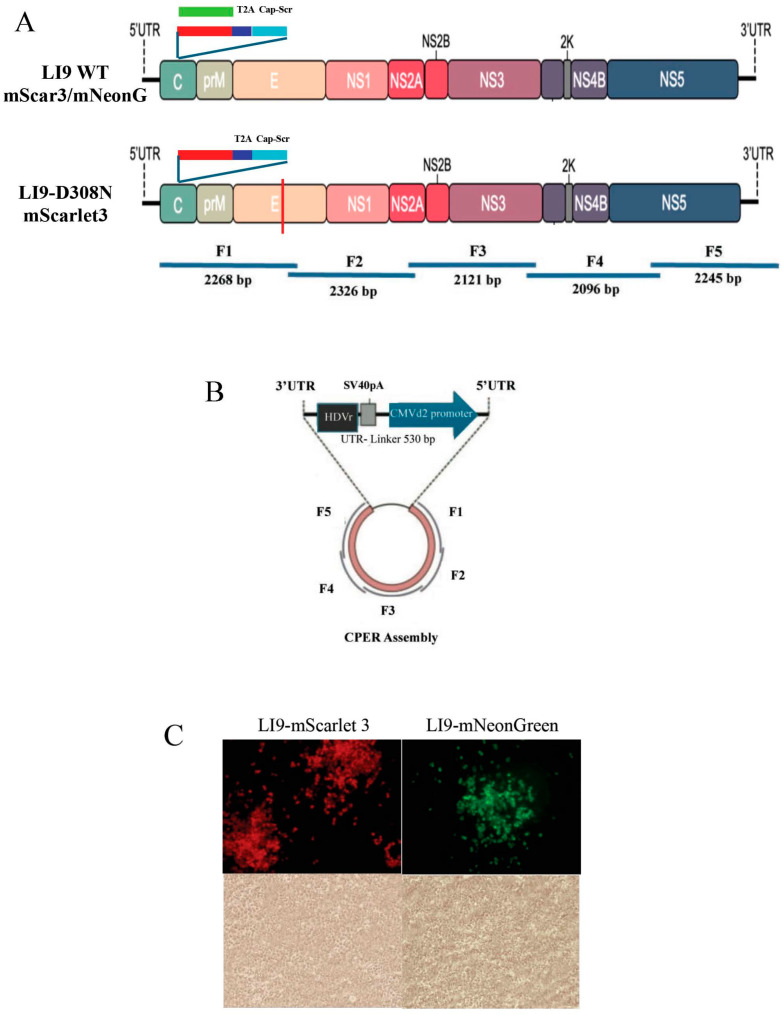
**CPER generation of recombinant fluorescent LI9 and LI9-D308N POWVs.** (**A**) Schematic of CPER-generated LI9 WT mScarlet3/mNeon and LI9-D308N mScarlet3 constructs. Reporter constructs were generated with a cassette of mScarlet3 (red) or mNeon (green) reporter genes followed by a T2A (blue) skipping motif and a scrambled capsid sequence (blue). (**B**) Schematic of CPER assembly construct containing overlapping fragments 1–5 and an LI9 3′ UTR linker containing the last 26 nucleotides of the 3′ UTR followed by a hepatitis delta virus ribozyme (HDVr), SV40 polyadenylation signal, a cytomegalovirus (CMV) promoter, and 33 bp of the LI9 5′UTR. (**C**) Representative fluorescence images (100×) of VeroE6 cells infected with LI9-mScarlet3 or LI9-mNeonGreen 7 days post-transfection.

Flaviviruses expressing fluorescent or enzymatic reporter proteins provide a rapid means of analyzing viral neutralization and cell tropism and screening antivirals. POWV LI9 strain, which was isolated from deer ticks in VeroE6 cells, spreads focally without cytopathology and causes highly lethal neurovirulence in aged immunocompetent B6 mice [29,30,31,54]. A robust LI9 POWV reverse genetics system was developed using CPER that bypasses stability issues of full-length genomic clones in bacteria [54]. In order to generate reporter POWVs, we CPER-assembled genetically modified LI9 POWVs that express fluorescent proteins from a set of overlapping LI9 cloned fragments (Figure 1A). POWV 5′ and 3′ untranslated regions (UTRs), along with initial capsid-encoding sequences, specify viral termini that are critical for viral replication. To produce reporter POWVs, a cassette containing enhanced fluorescent reporter proteins (mScarlet3 or mNeonGreen) [60,61], a T2A translational skipping motif (EGRGSLLTCGDVEENPG-P) [66,67] and a scrambled capsid sequence were inserted downstream of the 5′UTR and 38 native capsid residues in pMiniT clones of LI9 fragment 1 (F1) (Figure 1A). A linker comprising a CMVd2 promoter, a hepatitis delta ribozyme (HDVr) and an SV40 polyA addition site was used to circularize fragments 1 and 5 and produce authentic POWV termini (Figure 1B) [54]. Phusion polymerase-amplified fragments (F1–F5 and linker) were circularized prior to transfection of VeroE6 cells [54]. CPER-transfected VeroE6 cells produced fluorescent LI9-mScarlet3- or LI9-mNeonGreen-infected foci in 4–7 days (Figure 1C), with initial POWV reporter stocks (P0) collected 7–10 dpt. LI9-mScarlet3 and LI9–mNeonGreen reporter isolates that directed robust P0 VeroE6 cell fluorescence were further passaged in VeroE6 cells and sequence-verified.

### 3.2. LI9 Fluorescent (FL) Reporter Virus Replication Kinetics

**Figure 2:**
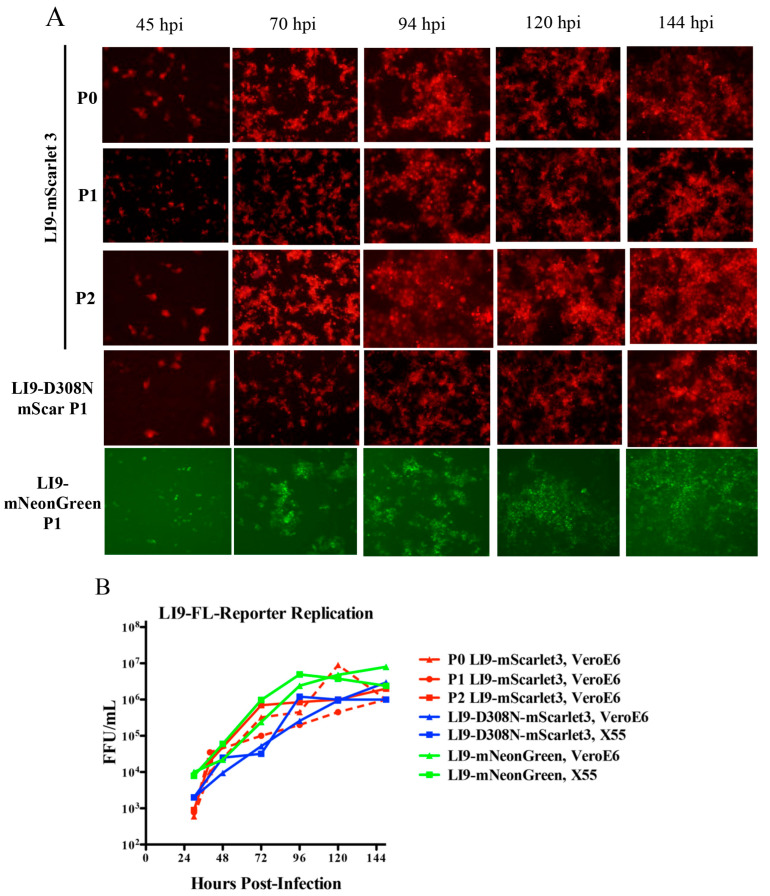
**Kinetic analysis of fluorescent reporter viruses.** Transfected reporter constructs produce infectious viruses that spread cell–cell, are stable across multiple passages, and replicate to similar titers. (**A**) Representative fluorescent images (100×) of cell–cell spread of passage 0, 1 and 2 viral stocks from LI9-mScarlet3 and Passage 1 viral stocks from LI9-mNeonGreen, and LI9-D308N-mScarlet3 in VeroE6 cells from 2–6 dpi. (**B**) Kinetics of fluorescent LI9 reporter virus replication in VeroE6 cells or NS1-expressing X55-VeroE6 cells. LI9-mScarlet3 P0, P1, P2 stocks grown on VeroE6 cells. LI9-D308N-mScarlet3 (MOI 0.1) or LI9-mNeonGreen (MOI 0.5) viruses were grown on VeroE6 and NS1-expressing X55-VeroE6 cells and supernatant titers assessed 1–6 dpi by focus assay. Data is presented as the mean +/− SD analyzed by two-way ANOVA. Titers were evaluated in duplicate and are representative of at least 2 experiments.

We previously reported that LI9 causes lethal age-dependent neurovirulence in mice and that mutating D308N in the POWV envelope protein abolished lethality [31]. Envelope D308N residues are exposed on the virion surface in a domain (EDIII) that is associated with OFV cell attachment and virulence [28,31,32,33,34,35,36,37,38,39,40]. Collectively, these findings suggest the potential for D308N mutations to alter cell attachment, systemic spread or neurotropism and thereby prevent lethal neuroinvasion. In order to investigate LI9-D308N cell tropism we generated a fluorescent LI9-D308N mutant reporter virus (Figure 1A and Figure 2A). LI9-mScarlet3, LI9-mNeonGreen and LI9-D308N-mScarlet3 viruses were sequence-verified, comparatively analyzed and stable in VeroE6 cells after sequential passage (P0, P1, P2) (Figure 2A). We limited our analysis of fluorescence to early-passage stocks based on reports of OFV reporter instability in later passages. Following LI9-FL reporter infections of VeroE6 cells, fluorescence was first observed at a single-cell level 24–32 hpi with subsequent non-lytic focal cell–cell spread (Figure 2A,B). Reporter fluorescence was observed in both the cytoplasm and nucleus, potentially reflecting the N-terminal fusion of 38 capsid residues to mScarlet3 and mNeonGreen. Reporter viruses replicated with at similar rates and to similar titers in both VeroE6 and NS1-expressing X55-VeroE6 cells (Figure 2B).

### 3.3. Generation of BSL2-Compatible LI9-ΔNS1 Fluorescent Reporter Viruses

**Figure 3:**
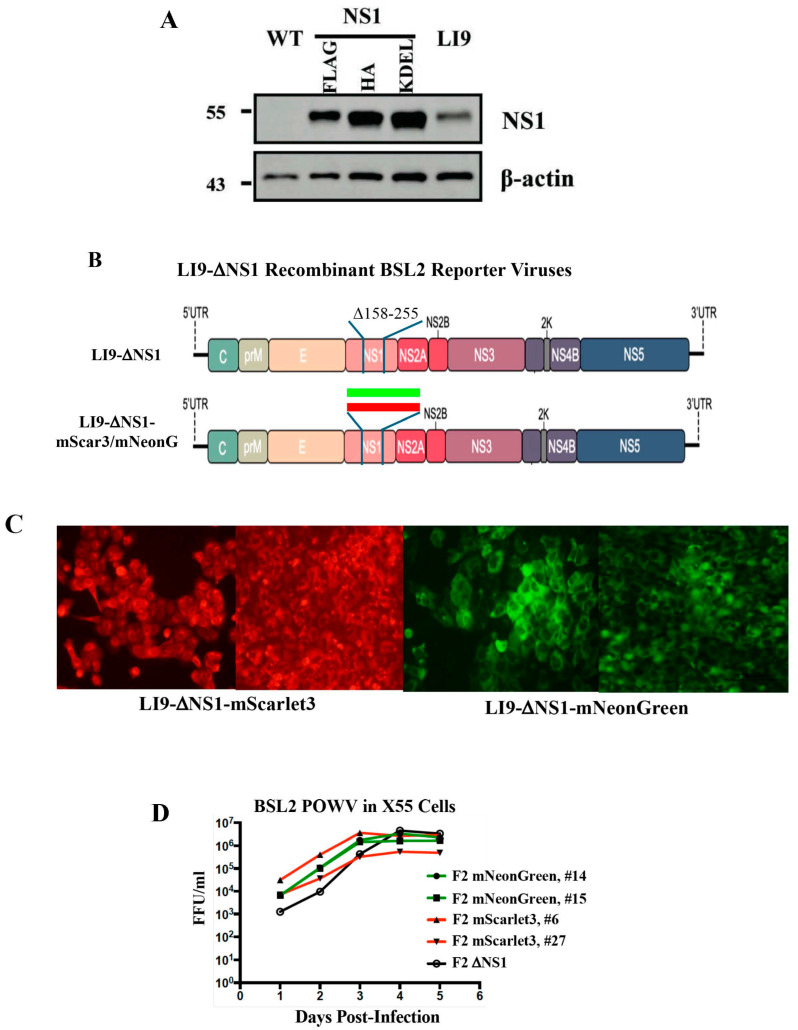
**Generation of NS1-expressing cell lines and LI9-ΔNS1-reporter POWVs.** (**A**) VeroE6 cells were transduced with lentiviruses expressing NS1-Flag, NS1-HA or NS1-KDEL, puromycin-selected and Western-blotted to assess NS1 expression. Expression levels were compared to NS1 protein levels in LI9-infected VeroE6 cells (MOI 1) harvested 4 dpi. (**B**) Schematic of CPER-constructed LI9-ΔNS1 with a 97 amino acid deletion in NS1 (blue lines) and the insertion of mScarlet3 or mNeonGreen into LI9-ΔNS1 virus (red and green bars) into NS1 deletion. (**C**) NS1-Flag-expressing VeroE6 cells were Lipofectamine 3000 transfected with CPER-assembled LI9-ΔNS1-mScarlet3 or LI9-ΔNS1-mNeonGreen DNAs. Representative images (100×) of fluorescent foci analyzed 7 days post-transfection. (**D**) CPER-generated stocks of LI9-ΔNS1-mScarlet3 (#6, #27) and LI9-ΔNS1-mNeonGreen (#14, #15) isolates and untagged LI9-ΔNS1, were assayed in duplicate for replication kinetics 1–5 dpi by quantitating fluorescent foci on NS1-Flag-expressing VeroE6 cells or by anti-POWV immunoperoxidase staining of LI9-ΔNS1-infected cells. Data is representative of at least 2 experiments and presented as the mean +/− SD analyzed via two-way ANOVA.

OFV NS1 proteins are translocated into the lumen of the ER, yet coordinate the assembly of cytoplasmic NS protein transcription complexes [1,23]. Deleting NS1 abolishes OFV replication, but expressing NS1 *in trans* has rescued the replication of DV, WNV and YFV NS1 deletion mutants and generated replication-defective viruses suitable for use in BSL2 settings and as vaccines [23,46,47,51,54,59]. To develop BSL2-compatible POWVs, we first generated VeroE6 cell lines constitutively expressing POWV NS1. Lentivirus plasmids were engineered to express NS1-Flag, NS1-HA and NS1-KDEL with C-terminal tags or ER retention motifs, as previously developed for DV [23]. VeroE6 cells were lentivirus-transduced, puromycin-selected (6 µg/mL) and analyzed by Western blot for NS1 protein expression [54]. In cell lines expressing POWV NS1-Flag, NS1-HA tags or NS1-KDEL ER-retention signals, NS1 levels exceeded expression directed by LI9 infection of VeroE6 cells (Figure 3A). Continuous passage and puromycin selection of VeroE6-NS1-Flag cells resulted in a novel integrated NS1-Flag cell line (X55-VeroE6) that constitutively expressed NS1 without further puromycin selection.

POWV with in-frame NS1-deletions (5–297 or 158–255) were generated in F2-containing plasmids and substituted for WT F2 fragments to generate LI9-ΔNS1 viruses (Figure 3B). Initial deletions of LI9 NS1 residues 5–297 failed to generate viable LI9-ΔNS1 viruses. In contrast, deleting 97 NS1 residues (158–255) in POWV LI9 successfully generated LI9-ΔNS1 viruses that replicated in all three NS1-expressing VeroE6 cell lines (Figure 3B). To insert reporter genes in NS1 deletions, F2 fragments with NS1 158–255 residue deletions were engineered to contain in-frame mScarlet3 or mNeonGreen reporters by HiFi DNA assembly (Figure 3B).

**Figure 4:**
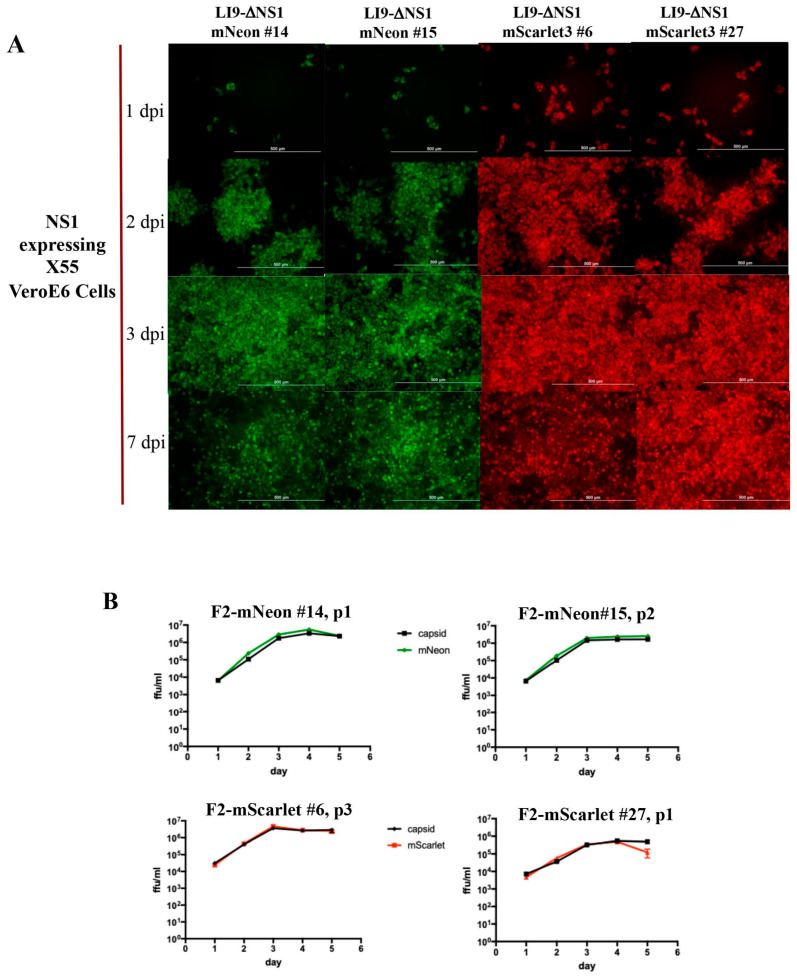
**Comparison of LI9-ΔNS1-reporter POWV isolates.** (**A**) NS1-expressing VeroE6 cells were infected with LI9-ΔNS1-mScarlet3 (#6, #27) or LI9-ΔNS1-mNeonGreen (#14, #15) isolates and infection and representative images (100×) of cell–cell spread was assessed 1–7 dpi by fluorescence microscopy. 1–7 dpi are presented. (**B**) Comparison of replication kinetics of LI9-ΔNS1-mScarlet3 (#6, #27) and LI9-ΔNS1-mNeonGreen (#14, #15) isolates 1–5 dpi in both NS1-expressing VeroE6 cells. Kinetics were determined by monitoring foci of infection by cell florescence (red, green) and comparatively by immunoperoxidase staining using an anti-capsid antibody, represented (black). Results reflect duplicate analysis of fluorescent and viral antigen determined titers with data presented as the mean +/− SD analyzed via two-way ANOVA.

CPER assembly and transfection of NS1-expressing X55-VeroE6 cells resulted in the production of LI9-ΔNS1 reporters that produced fluorescent foci following infection (Figure 3C). LI9-ΔNS1-reporter isolates replicated with similar kinetics and to high titers in NS1-expressing X55-VeroE6 cells (Figure 3D). Reporter fluorescence was observed in the cytoplasm and nucleus, potentially reflecting the N-terminal. Consistent with reporter insertion between NS1 residues 158 and 255, protein fluorescence was perinuclear and reflected ER translocation of NS1-reporter chimeras. Kinetic analysis of LI9-ΔNS1-mScarlet3 and LI9-ΔNS1-mNeonGreen viral isolates revealed that fluorescence was initially observed in single cells as early as ~24 hpi, progressed rapidly to infected foci by 48 hpi, and was widespread in monolayers of X55-VeroE6 cells 3–7 dpi without cytopathology (Figure 4A). Analysis of a series of POWV reporter isolates revealed nearly identical growth kinetics as comparatively quantitated by fluorescence and capsid protein immunoperoxidase staining of POWV-infected cells (Figure 4B). These findings demonstrate reporter virus stability by coincident fluorescence and viral protein expression in reporters across early passages (Figure 4B). CPER isolates of LI9-ΔNS1-mNeon (#14, #15) and LI9-ΔNS1-mScarlet3 (#6, #27) replicated in X55-NS1-expressing VeroE6 cells, but failed to infect VeroE6 cells without expressed NS1 at any viral passage (Figure S1). To evaluate reversion of LI9-ΔNS1-reporter virus to WT, we passaged LI9-ΔNS1-reporter virus isolates in VeroE6 cells and found no infected cells at passage 5 or passage 10 (Figure S1). These findings validate that *in trans* NS1 expression is required for LI9-ΔNS1-reporter replication and demonstrate the absence of LI9-ΔNS1-reporter reversion to WT POWV over time and passage.

### 3.4. Effect of NS1 Expression and D308N Mutations on LI9 Reporter Replication

**Figure 5:**
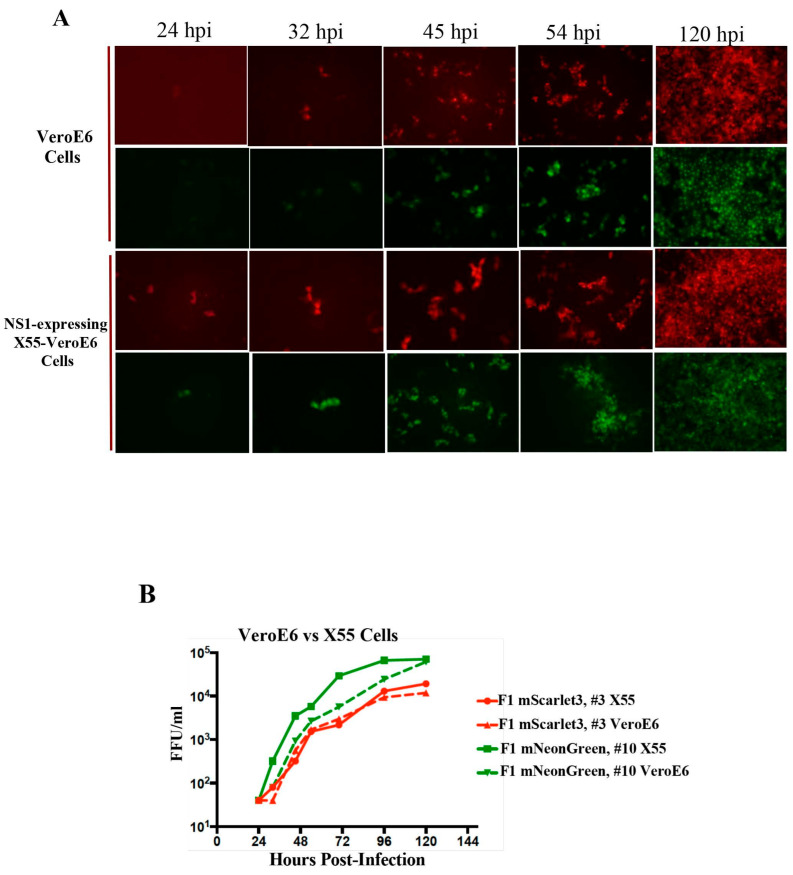
**Analysis of BSL3 POWV reporter replication in NS1-expressing cells.** (**A**) Comparison of cell–cell spread of LI9-mScarlet3 and LI9-mNeonGreen in VeroE6 versus X55-VeroE6 cells 1–5 dpi. VeroE6 cells and X55-VeroE6 cells were infected (MOI 0.1) with LI9-mScarlet3 and LI9-mNeonGreen and assessed by fluorescence microscopy (100×) 1–5 dpi. (**B**) Comparison of replication kinetics of LI9-mScarlet3 and LI9-mNeonGreen in both NS1-Flag-expressing VeroE6 cells and VeroE6 cells. Titers were assessed by quantitating fluorescent foci 1–5 dpi. Results of duplicates were determined by quantitating infected fluorescent cells and presented as the mean +/− SD analyzed via two-way ANOVA.

**Figure 6:**
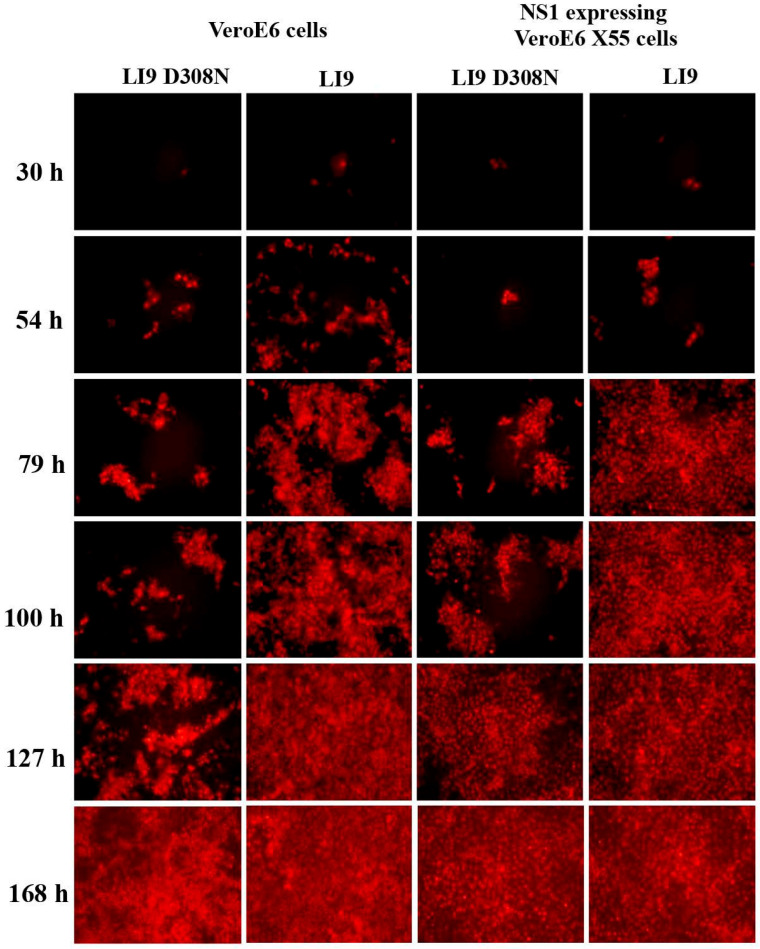
**Avirulent LI9-D308N mutant spreads cell-to-cell in NS1-expressing VeroE6 cells**. VeroE6 and NS1-Flag-expressing VeroE6 cells were infected with LI9-mScarlet3 and LI9-D308N-mScarlet3 (MOI 0.1) and assessed by fluorescence microscopy. Representative images of focal POWV reporter spread (100×) are presented.

The rapid fluorescence of LI9-ΔNS1-reporters in NS1-expressing cells suggested the potential for *in trans* NS1 expression to enhance the spread and titer of fluorescent BSL3 LI9-reporter viruses. A comparison of LI9-mScarlet3 and LI9-mNeonGreen replication in VeroE6 cells versus NS1-expressing VeroE6 cells revealed comparable replication and spread of BSL3 LI9 reporter viruses in both cell lines 1–5 dpi (Figure 5A,B). Similar comparisons of LI9-mScarlet3 and LI9-D308N-mScarlet3 virus replication in VeroE6 cells, vs. NS1-expressing VeroE6 cells, indicates that NS1 expression has no apparent effect on the kinetics of BSL3 POWV reporter virus spread (Figure 6).

### 3.5. LI9 and D308N Reporter Virus Replication and Spread in CNS Cell Lines

**Figure 7:**
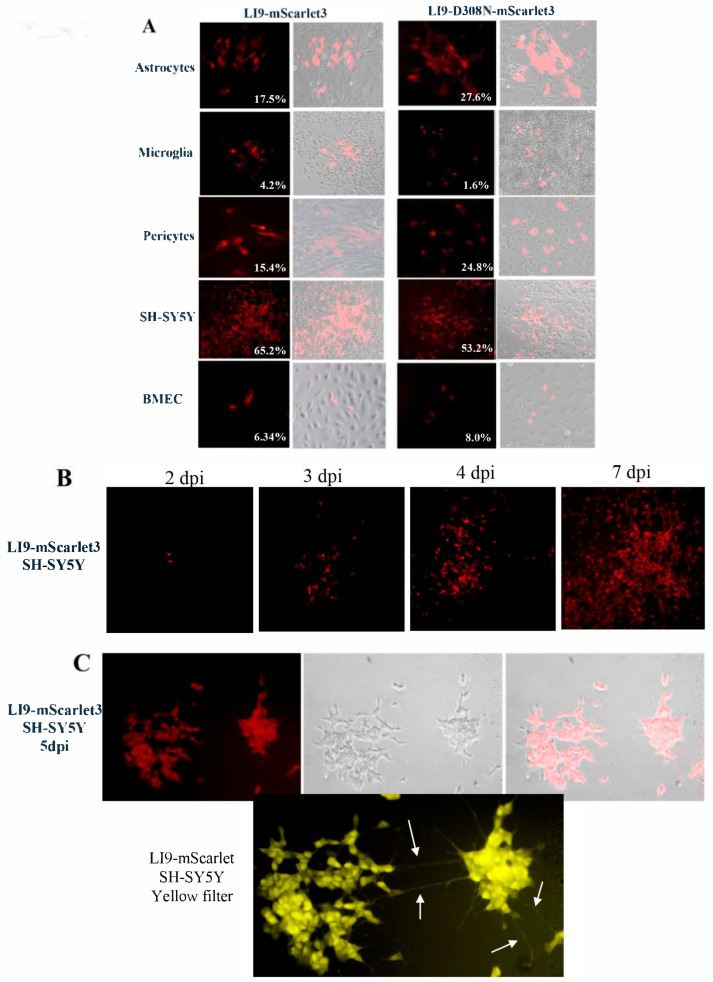
**LI9-mScarlet3 and LI9-D308N-mScarlet comparably infect astrocytes, microglia, pericytes, neuronal-like SH-SY5Y cells and hBMECs in vitro**. (**A**) Neuronal-like cells (SH-SY5Y), astrocytes (C8-D1A), microglia (SIM-A9), pericytes (HVEP) and human-brain endothelial cells (hBMECs) were infected with LI9-mScarlet3 and LI9-D308N mScarlet at (MOI 1) and representative images of infection between days 3–7 are presented (200×). The white number indicates the approximate percentage of susceptible cells determined by manual counting of at least 5000 cells total from LI9-mScarlet infections and 3500 cells total from LI9-D308N-mScarlet infections. (**B**) Representative images of LI9-mScarlet3 infection (MOI 1.0) and kinetic focal cell-to-cell spread in differentiated SH-SY5Y cells was assessed 2–7 dpi. (**C**) Representative images of LI9-mScarlet3 infection of differentiated SH-SY5Y neuronal cells using red and yellow filters reveal fluorescent neurite projections (white arrows) connecting groups of SH-SY5Y cells (200×). Data is representative of at least 2 independent experiments.

The envelope protein EDIII domain is associated with OFV cell attachment and attenuation [4,28,31,32,33,34,35,36,37,38,39,40,41,42,43,44,45]. In B6 mice, LI9-D308N mutations prevent neurologic symptoms, neuropathology and CNS neuroinvasion [29,31], however, whether LI9-D308N EDIII mutations prevent viral attachment and entry into the blood–brain barrier or CNS cells remains unknown. As a potential mechanism of POWV neuroinvasion, we previously found that human blood–brain-barrier-associated pericytes and brain vascular endothelial cells (hBMECs) are persistently infected by LI9 and that POWV spreads basolaterally across hBMECs [30]. Here, we show that like WT POWV LI9, LI9-mScarlet3 reporter viruses infected hBMECs and pericytes, astrocytes (C8-D1A), microglia (SIM-A9) and differentiated SH-SY5Y neuron-like cells (Figure 7A) [62,63,64]. Similar to LI9-mScarlet3 infection of CNS cells, we found that the LI9-D308N-mScarlet3 mutant comparably infected BBB and CNS cell types in vitro (Figure 7A). Notably, LI9-mScarlet3 reporters were found to persistently infect differentiated SH-SY5Y cells (>7 dpi) reaching titers of ~2 × 10^5^ FFU/mL without apparent cytopathic effects. Differentiation of SH-SY5Y cells with retinoic acid (RA) and brain-derived neurotrophic factor (BDNF), results in SH-SY5Y neurite extensions that mediate neuronal connections in vitro [62,63,64]. Following infection of differentiated SH-SY5Y cells with LI9-mScarlet3, neurites displayed clearly visible fluorescent cell–cell extensions that stand out in the yellow filter image in Figure 7C, suggesting the potential for cell-to-cell spread. Overall, these in vitro findings suggest that failure of LI9-D308N to enter the CNS and infect CNS cells is not likely to be determined by altered viral attachment and entry into BBB or CNS cells.

## 4. Discussion

POWVs are present in ~2% of *Ixodes scapularis* ticks (deer ticks) on Long Island, NY [11] and following transmission cause lethal encephalitis (~10%) and long-term neurologic sequelae in ~50% of survivors [2,17,19]. Currently there is little understanding of POWV systemic dissemination, neuroinvasion or roles for immune-cell recruitment and CNS cell responses in neuropathogenesis. Studying POWVs in BSL3 and ABSL3 settings complicates experiments defining pathogenic mechanisms. Fluorescent reporter viruses have proven to be useful tools for assessing viral kinetics, neutralization and antivirals and for evaluating cellular targets in BSL2 settings [50,51,52,58]. Here, we used reverse genetics to generate novel BSL2 and BSL3 fluorescent reporter POWVs that replicate to high titers and are stable through multiple passages. Infection of cells with LI9 or LI9-D308N reporter viruses resulted in robust fluorescence that mirrored the focal, non-lytic, cell-to-cell spread of WT LI9 POWV reporters. As a result, POWV reporters provide a means for analyzing POWV spread and cell and tissue tropism and for defining determinants of POWV virulence and attenuation in vitro and, potentially, in vivo.

Replication-defective surrogate viruses have been generated for many BSL3 and BSL4 viruses in order to permit experimentation in less restrictive biosafety facilities [50,51,52,58]. In order to create BSL2 POWVs, we produced VeroE6 cells that express POWV NS1 and used these cell lines to rescue replication-defective NS1-deleted LI9 viruses. POWV-ΔNS1-FL reporter viruses spread focally, are non-lytic, replicate to high titers and direct rapid fluorescence in infected VeroE6-NS1 cells. Consistent with a 97-residue NS1 deletion, repeated passage of POWV-ΔNS1-FL reporters in VeroE6 cells lacking NS1 failed to produce WT revertants. As replication-defective OFVs have been proposed as potential vaccines [46,47,68,69], POWV-ΔNS1-FL reporter viruses may provide a means of inducing protective immune responses and rationalize assessing POWV-ΔNS1 viruses as nonreplicating vaccine candidates.

POWVs and TBEVs are both neuroinvasive OFVs that result in lethal outcomes and long-term neuropathology [1,2,3,4,5,6]. TBEV neuropathology is characterized by meningitis with perivascular cuffing and polioencephalomyelitis of the brain and spinal cord, accompanied by neuronal degradation [2,70,71,72]. TBEVs infect neurons and Purkinje cells in the human CNS and in murine TBEV infection models [73,74,75,76,77,78]. In addition, analysis of TBEV-infected cell lines in vitro, and i.c. inoculation of a fluorescent TBEV reporter into murine brains demonstrate that neurons, pericytes, microglia and astrocytes are susceptible to TBEV infection [56,58,73,74,79,80,81,82,83,84].

POWV LI9 enters the CNS causing neuropathology and age-dependent lethality in immunocompetent mice [29]. POWV encephalitis is characterized by spongiform lesions and glial cell activation in the absence of perivascular cuffing [29,30,31,54]. In contrast to LI9, avirulent POWV mutants, LI9P and LI9-D308N, fail to enter the CNS or cause CNS pathology or lethality, yet elicit immune responses that protect mice from a lethal challenge [31]. Both avirulent POWVs contain D308N mutations in envelope protein EDIII domains that are associated with OFV cell attachment and virulence [48,49,50,51,52,53,54]. The role of D308N in regulating CNS entry prompted us to determine whether the susceptibility of CNS cells to LI9-D308N virus infection is a determinant of restricted neuroinvasion. Using fluorescent reporter POWVs our findings demonstrate that WT LI9-mScarlet3 and mutant LI9-D308N-mScarlet3 viruses are both able to infect astrocytes, microglia, differentiated neuron-like (SH-SY5Y) cell lines [62,63,64], primary human pericytes and hBMECs in vitro. As a result, in vitro differences in LI9-D308N cell attachment and entry into CNS cells are not apparent from our analysis, and the mechanism of failed neuroinvasion by LI9P and LI9-D308N viruses remains an enigma.

Several CNS entry mechanisms have been reported for neurovirulent WNVs and JEVs to enter the CNS including retrograde transport, immune cell transmigration and BBB permeability [85,86,87,88,89,90,91,92,93,94,95]. However, these OFVs are also associated with meningitis and perivascular cuffing consistent with a hematogenous route of CNS entry, that is not observed following POWV LI9 infection [89,92,93,96,97,98,99,100]. Our findings suggest that D308N mutations impact alternative routes of POWV entry into the CNS (axonal, immune cell, CSF), immune clearance from the blood or charge-dependent interactions resulting from removing 180 aspartic acids from the virion surface. Whether POWV D308N mutations alter discrete CNS routes of entry or systemic spread remains to be investigated.

Our results demonstrate the ability of POWVs to infect neuronal SH-SY5Y cells [62,63,64], microglia, astrocytes and human-brain vascular pericytes and microvascular endothelial cells [22,76,101]. Our findings are consistent with cell susceptibility to TBEV, although the approximate percentages of susceptible cells differ [82,83,84]. Susceptibility differences may stem from prior TBEV neuroadaptation in suckling mouse brains as well as the use of different cell lines, MOIs and days of post-infection analysis [82,83,84]. LI9-mScarlet3 infection of differentiated SH-SY5Y cells revealed fluorescence within neurite extensions (Figure 7C) suggesting the potential for transport of POWV-infected cytoplasm to adjacent neurons without immune surveillance. Whether LI9 infection of neurons results in focal cell-to-cell spread in the presence of neutralizing antibodies, and mirrors LI9 infection of VeroE6 cells, remains to be explored.

This study has generated novel fluorescent reporter POWVs that permit analysis of cellular targets, focal cell-to-cell POWV spread and determinants of virulence in BSL3 and BSL2 settings. Fluorescent POWV reporters allow real-time live cell imaging of viral entry and spread that is a fundamental advantage over WT POWV infections that require fixation and staining. Importantly we found that POWV-LI9 reporters infect hBMECs, pericytes, astrocytes, microglia and neuronal SH-SY5Y cell types that suggest cell targeting and spread within the CNS following POWV neuroinvasion. Our findings further demonstrate that LI9-D308N mutations do not restrict the infection of CNS cells and rationalize analyzing peripheral viral clearance and alternative CNS entry routes for roles in restricting LI9-D308N neuroinvasion. Overall our fluorescent POWV reporters provide the means to define determinants of POWV pathogenesis and for replication-incompetent POWVs to be evaluated as potential vaccines.

## Data Availability

The methods underlying this study are available in an online supplement from the authors’ by request.

## Supplementary Materials

The following supporting information can be downloaded at: https://www.mdpi.com/article/10.3390/v18070768/s1, Figure S1: No reversion of LI9-ΔNS1-mScarlet3 or LI9-ΔNS1mNeonGreen viruses after passage.
