# Supplementary material for: Fluorescent Powassan Reporter Viruses Infect Neuron, Astrocyte and Microglial Cell Lines Independent of Attenuating D308N Envelope Protein Modification
Source: Viruses. 2026 Jul 13;18(7):768. doi: 10.3390/v18070768 (PMC13431600; doi:10.3390/v18070768)
Supplement: Supplementary file 1 [file viruses-18-00768-s001.zip › viruses-4348446-supplementary.pdf]

Figure S1

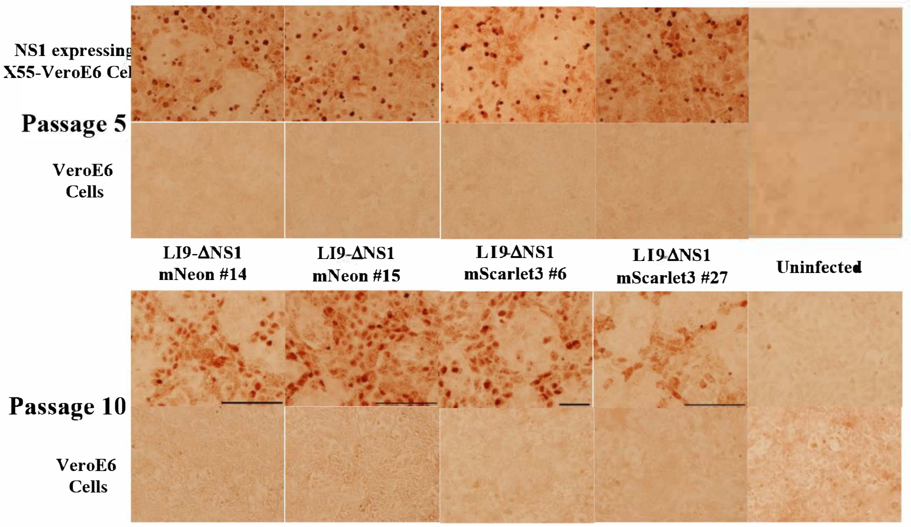

Figure S1. No reversion of LI9-ΔNS1-mScarlet3 or LI9-ΔNS1mNeonGreenviruses after passage.

Vero E6 cells, or VeroE6 cells stably expressing POWV NS1 protein, were plated in 24-well plates and infected with Powassan viruses, lacking NS1, at MOI 1 (passage 1). In 3 to 4 days, freshly plated NS1 expressing cells were inoculated with supernatants from passage 1 (passage 2). The viruses were passaged 10 times in this manner. At passages 5 and 10, wild type Vero E6 cells were inoculated with the same supernatants as NS1 expressing cells. The plates were fixed with 100% methanol and stained with POWV LI9 capsid antibody and visualized at 20×. Scale bar 100 µm.
